# *Culex*-Transmitted Diseases: Mechanisms, Impact, and Future Control Strategies using *Wolbachia*

**DOI:** 10.3390/v16071134

**Published:** 2024-07-15

**Authors:** Mukund Madhav, Kim R. Blasdell, Brendan Trewin, Prasad N. Paradkar, Adam J. López-Denman

**Affiliations:** 1 Australian Centre for Disease Preparedness, CSIRO Health and Biosecurity, Geelong, VIC 3220, Australia; 2 CSIRO Health and Biosecurity, Dutton Park, Brisbane, QLD 4102, Australia

**Keywords:** *Culex*, *Wolbachia*, arboviruses, mosquito control

## Abstract

Mosquitoes of the *Culex* genus are responsible for a large burden of zoonotic virus transmission globally. Collectively, they play a significant role in the transmission of medically significant diseases such as Japanese encephalitis virus and West Nile virus. Climate change, global trade, habitat transformation and increased urbanisation are leading to the establishment of *Culex* mosquitoes in new geographical regions. These novel mosquito incursions are intensifying concerns about the emergence of *Culex*-transmitted diseases and outbreaks in previously unaffected areas. New mosquito control methods are currently being developed and deployed globally. Understanding the complex interaction between pathogens and mosquitoes is essential for developing new control strategies for *Culex* species mosquitoes. This article reviews the role of *Culex* mosquitos as vectors of zoonotic disease, discussing the transmission of viruses across different species, and the potential use of *Wolbachia* technologies to control disease spread. By leveraging the insights gained from recent successful field trials of *Wolbachia* against *Aedes*-borne diseases, we comprehensively discuss the feasibility of using this technique to control *Culex* mosquitoes and the potential for the development of next generational *Wolbachia*-based control methods.

## 1. Introduction

With their potential for spillover into human populations, zoonotic vector-borne diseases pose significant public health challenges [1]. Over the past two decades, numerous vector-borne pathogens have expanded into new geographic regions, while endemic diseases have surged in prevalence [2]. Mosquitoes from the genus *Culex* hold substantial epidemiological importance among the vectors involved in transmitting such diseases. *Culex* mosquitoes spread medically relevant pathogens like filarial parasites and arthropod-borne viruses (arboviruses), resulting in morbidity and mortality in tropical and subtropical areas. To date, various control strategies have been used to prevent the prevalence of *Culex*-borne diseases, including behavioural, chemical, and biological interventions. However, existing control strategies have a number of challenges and varying degrees of success. The inefficiency of the existing conventional method, combined with climate change and urbanisation, is leading to increased interactions between *Culex* and humans, resulting in global outbreaks of *Culex*-borne diseases in new areas.

*Wolbachia* has emerged as a potential method to combat mosquito-borne disease in the last decade. *Wolbachia* is an intracellular endosymbiont bacterium of arthropods and nematodes, which often manipulates host reproduction and/or blocks transmission of arboviruses such as dengue, Zika and chikungunya. The most common reproductive manipulation induced by *Wolbachia* is cytoplasmic incompatibility (CI). CI occurs when the viability of embryos is reduced due to the mating of *Wolbachia*-infected males with uninfected females. In some hosts, other *Wolbachia*-induced reproductive manipulation may include parthenogenesis (development of offspring from unfertilised eggs), feminisation (conversion of genetic males into females), or male killing (death of male embryos) [3]. *Wolbachia* is a maternally transmitted endosymbiont, and all these reproductive manipulations ensure that a higher proportion of females are infected to maintain *Wolbachia* infection within the host population [4]. Field trials of *Wolbachia* involve either suppressing the mosquito population by releasing *Wolbachia*-infected males or replacing the targeted mosquito population with *Wolbachia*-infected males and females. In population replacement, the newly introduced *Wolbachia*-infected mosquito in the population will have a reduced ability to transmit dengue viruses (see Section 7 and Section 9). In order to suppress the transmission of dengue virus in the community, mosquitoes infected with *Wolbachia* are being released in various countries, including the USA [5,6], Brazil [7,8], Italy [6], Australia [9], Vietnam [10], Indonesia [11,12], Singapore [10], China [13] and Malaysia [14]. *Wolbachia* trials have demonstrated significant reductions in dengue incidence following the release of mosquitoes infected with *Wolbachia*, with virus suppression rates ranging from 40% in Kuala Lumpur, Malaysia [14], to over 70% in Yogyakarta, Indonesia [15] and 96% in northern Queensland, Australia [7]. Encouraging findings from *Wolbachia* trials highlight the potential of *Wolbachia*-based strategies as a sustainable and environmentally friendly approach to control mosquito-borne diseases on a global scale.

This review attempts to give a comprehensive overview of the current understanding of *Culex* mosquitoes as vectors of viral zoonotic diseases in humans and insight into their molecular interaction with viruses. It also summarises what has been achieved so far in the field regarding the use of *Wolbachia* to control *Culex* mosquitoes and how this approach can be further fine-tuned for future use.

## 2. The *Culex* Mosquito Lifecycle and Its Role in Pathogen Transmission

The *Culex* genus of mosquitoes encompasses numerous medically significant mosquito species responsible for spreading various human and animal pathogens globally [16]. The mosquito life cycle consists of four distinct stages: egg, larvae, pupae and adult. While the cycle duration can vary slightly between species, it typically spans 2–4 weeks and is influenced by environmental conditions such as temperature [17,18]. The larval and pupal life stages are exclusively aquatic, with adult mosquitoes emerging from the water to initiate a new reproductive cycle. Unlike *Aedes* spp., female *Culex* mosquitoes deposit their eggs in specialised rafts, grouping them together to float on the surface of both fresh and stagnant water. *Culex* mosquitoes oviposit significantly more eggs compared to *Aedes* spp. mosquitoes, and as such, local populations can rapidly expand under optimal conditions. Oviposition sites range from natural habitats to diverse locations such as puddles, drains, ditches, or even tin cans, with larvae emerging within 24–48 h post-laying [19].

Similar to other hematophagous mosquito species, female *Culex* mosquitoes are compelled to seek out a blood meal to facilitate egg production, presenting a potential avenue for pathogen transmission. The choice of hosts plays a major role in pathogen spread and differs between *Culex* species [20,21,22,23,24]. Factors such as time of year, host availability, and transmission dynamics are closely intertwined with local ecology and climate variation throughout the year, influencing mosquito blood-feeding habits [20,25,26,27]. Additionally, intricate interactions among viruses, mosquito vectors, and hosts can further determine the severity and significance of disease outbreaks.

Among the most significant species include *Culex pipiens* and *Culex quinquefasciatus*, given their ability to transmit multiple viruses and their opportunistic feeding on both human and animal hosts [28,29,30]. This dual-feeding behaviour amplifies the public health risk, contributing to the potential spread of zoonotic diseases and outbreaks of mosquito-borne pathogens [16,31]. However, focusing solely on these mosquitoes presents a challenge, as the distribution of other *Culex* species poses region-specific challenges in pathogen transmission (Table 1).

**Table 1:** *Culex* mosquito species and the pathogens they transmit.

Mosquito	Pathogen(s) They Transmit
***Cx. annulirostris***	JEV [32], Murray Valley encephalitis virus (MVEV) [33], Ross River virus (RRV) [34], Barmah Forest Virus [35]
***Cx. australicus***	MVEV [34], WNV [36]
***Cx. erraticus***	EEEV [37], WNV [38]
***Cx. gelidus***	RRV, JEV, WNV, MVEV [39]
***Cx. modestus***	WNV, Usutu [40]
***Cx. pipiens***	WEEV, WNV, JEV [41,42,43], Avian Plasmodium [26]
***Cx. quinquefaciatus***	SLEV [44], WNV [45] Avian Plasmodium [46]
***Cx. restuans***	WNV [47]
***Cx. tarsalis***	WNV [48], Cache valley virus [49], Rift Valley fever virus (RVFV) [50], WEEV, SLEV [51]
***Cx. territans***	*Batrachochytrium dendrobatidis* [52]
***Cx. theileri***	Dirofilariasis [53], WNV [54], RVFV [55], Avian plasmodium [56]
***Cx. tritaeniorhynchus***	JEV [57], CQV [58], WNV [59]

*Culex* mosquitoes can transmit multiple medically significant arboviruses, including Japanese encephalitis virus (JEV) [43], West Nile virus (WNV) [42], Usutu virus (USUV), St. Louis encephalitis virus (SLEV) [44], Western and Eastern equine encephalitis viruses (WEEV/EEEV) [33,34] and Cat Que virus (CQV) [37,41]. Additionally, *Culex* mosquitoes can vector parasites such as nematodes responsible for lymphatic filariasis and protists responsible for avian malaria [60,61,62]. The transmission dynamics of these pathogens are highly dependent on the local distribution of specific *Culex* species, leading to distinct transmission risk factors dependent on geographical location [63].

While mosquitoes primarily transmit pathogens by ingesting an infected blood meal, incubating these pathogens internally and then transmitting the disease-causing agents to a vertebrate host, *Culex* spp. mosquitoes have also been implicated in the mechanical transmission of pathogens [52,64]. Mechanical transmission involves transferring an infectious agent to a new host through direct contact with the mosquitos’ mouthparts, legs, or body. Although understanding mechanical transmission is crucial for understanding virus transmission dynamics, its significance in *Culex* mosquitoes remains poorly understood and requires further assessment.

There are two main concepts that are important in disease transmission by mosquito vectors: “Vector competence” and “vectorial capacity”. Vector competence describes a mosquito’s ability to acquire, maintain, and subsequently transmit a specific pathogen. “Vectorial capacity” is a measure of the transmission potential of a pathogen within a population and includes external factors such as mosquito behaviour, survival, and pathogen biology. The capability to transmit a pathogen is contingent on various factors, including species and environmental conditions. These factors are critically important in understanding vector biology and the epidemiology of viruses, and the impact they can have in disease maintenance and transmission. It is important to note that not all mosquitoes exhibit the same competence as vectors, and analysing vector competence of individual species can inform on the local distribution of pathogens and the potential for exotic pathogens to spread. Understanding individual vector competence is critical for discerning the necessary response to novel pathogen incursion and designing mosquito control programs. Moreover, understanding individual species vector competence plays a key role in informing effective public health responses to potential future epidemics based on mosquito species’ presence in an area.

## 3. Molecular and Cellular Interactions between *Culex* Mosquitoes and Viruses

Molecular and cellular virus-mosquito interactions during infection and transmission involve a series of complex processes within various mosquito tissues. Upon the uptake of a viraemic blood meal, the virus initially resides within the mosquito midgut. The first barrier viruses must overcome is infecting the cells of the midgut, achieved through receptor-mediated endocytosis, and is crucial for subsequent viral dissemination [65,66,67,68]. At this stage, a delicate balance exists between viral replication and local immunity, where viral replication may be overcome by the first stages of the mosquito immune response [69,70]. If successful in overcoming this barrier, viruses encounter the second barrier, escaping the midgut through the basal lamina, and disseminate to secondary tissues, such as the salivary gland, brain, and extremities, via the haemolymph [45]. Infection of the salivary gland is crucial for further viral dissemination, as the release of virions into the saliva is necessary for future transmission events [71]. However, viral replication is not always guaranteed, and the mosquito immune system is multifaceted and capable of mounting an effective antiviral response.

As viruses navigate through each defensive barrier within the mosquito, they encounter a robust array of immune responses mounted by the mosquito itself. Unlike mammals, mosquitoes lack an adaptive immune response and depend on innate immunity to defend against viruses. On sensing a virus, innate immune pathways are activated, either in a cellular or humoral manner. The humoral response activates downstream signalling pathways culminating in the production and secretion of effector molecules, such as antimicrobial peptides (AMPs) and phenoloxidases, into the haemolymph [72,73]. These responses can be initiated by various signalling cascades, including the JAK/STAT, Toll, and immune deficiency (Imd) pathways [69,74,75,76]. While these pathways are important in modulating immune defence, the most significant antiviral response in mosquitoes is the RNA interference (RNAi) pathway [77,78]. Recognition of viral RNA occurs through an interplay between pathogen recognition receptors (PRRs) interacting with pathogen-associated molecular patterns (PAMPs). This leads to the generation of small RNAs produced by the RNAi pathway to target viruses for degradation directly [65,78,79].

The cellular immune response is primarily mediated by a group of cells known as haemocytes that are present within the mosquito haemolymph. Operating similarly to mammalian macrophages, haemocytes can mount a multimodal immune response and are capable of encapsulating and phagocytosing pathogens [72,80,81]. Haemocytes can also contribute to the production of AMPs and phenoloxidases to further target infectious agents within the mosquito. In addition to these immune pathways, the cellular process of autophagy has been shown to be a significant mechanism that provides a kind of antiviral immunity by tagging infected sub-cellular components for degradation [81,82,83]. This pathway works by the creation of autophagosomes that can encapsulate damaged organelles or misfolded proteins, facilitating their degradation with the lysosome. This process aids by targeting active virus replication and eliminating the compromised cellular components.

Whilst mosquitoes are vectors for a significant number of pathogens, they exhibit a comprehensive array of antiviral responses to combat infection. By unravelling the intricacies of these mechanisms, there is the potential to devise methods for manipulating the mosquito immune system to develop control strategies within these insects. It is important to note that variation exists among mosquito species and to fully understand how these processes work in *Culex* mosquitoes, more thorough investigations are required.

## 4. Impact of Climate Change, Habitat Alterations, and Urbanisation on *Culex*-Transmitted Diseases

Major human-driven processes affecting the world today, climate change, urbanisation and globalisation, are influencing the distribution of *Culex* spp. and the pathogens they carry. Several *Culex* spp. can be found in urban environments, including *Cx. annulirostris*, *Cx. pipiens, Cx. quinquefasciatus and Cx. tritaeniorhynchus* [84,85,86]. *Cx. quinquefasciatus,* in particular, appears to thrive in urban areas, showing rapid colonisation of newly urbanised zones as seen after the catastrophic 2010 earthquake in Haiti [87]. While some forms of *Cx. pipiens* demonstrate population declines in urban areas [88], in temperate climates, the *Cx. molestus* form has become specialised to particular urban environments, showing a preference for subterranean habitats, including sewers, basements and underground rail lines [26,89]. Rising levels of urbanisation are, therefore, likely to increase the availability of suitable habitats for at least some *Culex* species and, in turn, the distribution of their pathogens.

Climate change is likely to expand the distribution and activity of many major vector species. Modelling studies have predicted range expansions *for Cx. pipiens, Cx. quinquefasciatus* and *Cx. tritaeniorhynchus* in a warming world [90,91,92]. Experiments assessing development and survival have found that warmer temperatures sped up *Culex* larval development; however, larval and adult survival decreased above certain temperatures [18,93]. This suggests that although a warming climate may be beneficial for pathogen transmission in some areas, through the facilitation of longer transmission seasons, transmission may actually be inhibited in other areas of greater temperature rise.

Both climate change and globalisation have likely facilitated the geographic expansion exhibited by several significant *Culex*-borne viruses in recent years. The most dramatic global expansion has been observed for WNV. This virus spread rapidly throughout North America following an initial incursion in 1999 and has also caused regular outbreaks in Europe over the last two decades [94]. Originally detected in Uganda in 1937, WNV now occurs commonly on every continent except Antarctica, with its global prevalence significantly increasing in recent years [94,95,96,97,98,99,100,101]. The range of JEV has also recently increased, with evidence of genotype replacement observed in some areas [102]. Expansion into Europe was confirmed in 2010 through molecular detections of JEV in *Cx. pipiens* mosquitoes and passerine birds from Italy [103,104], whilst a human case of JEV in Angola in 2016 represented the first autochthonous evidence of JEV in Africa [105]. In Australia, occasional small-scale outbreaks of JEV have been detected since 1995 [106], but the first widespread and most southern-latitude outbreak occurred in 2022. This outbreak affected multiple states, causing 46 human cases and 7 deaths, with evidence of infection detected in 80 piggeries, sentinel chickens and mosquitoes [32,107,108]. This virus is now considered likely endemic in parts of Australia, although very little is known about this endemic cycle. Other *Culex*-associated pathogens with evidence of geographic range expansion include the continued northward spread of EEEV in North America [109], Usutu virus, which is now considered endemic in parts of Europe [110], and avian malaria, which is expected to continue spreading in both Europe and North America while threatening native birds with extinction across the Pacific [111,112].

The likelihood of virus transmission requires susceptible vertebrate hosts along with a competent mosquito vector. Globalisation has resulted in the widespread introduction of several livestock and pest species, presenting opportunities for the establishment of geographically expanding pathogens that utilise these species. For example, pigs (*Sus scrofa*) are found on all continents except mainland Antarctica [113] and are also a significant amplifying host for JEV. In Australia, the first detection of JEV during the large 2022 outbreak was in commercial piggeries, but infections were also detected in feral pigs [32]. Australia plays host to an estimated population of several million largely unregulated feral pigs, representing a substantial potential host pool for this virus [114].

Alongside the utilisation of traditional host species, pathogen spread to new areas provides opportunities for infection of and adaptation to new hosts. When WNV entered new ecosystems in North America, it rapidly infected a range of previously unexposed avian species, resulting in significant mortality events in some species, such as the American crow (*Corvus brachyrhynchos*) [115,116]. This was subsequently linked to a single amino acid change in the NS3 protein, causing increased viral replication and transmissibility [117]. Usutu virus has also been linked to mortality events in blackbirds (*Turdus merula*) during the pathogen’s spread through Europe [118]. Interestingly, adaptation to new avian hosts has led to speciation events in avian malarial parasites [119]. Finally, the density of suitable hosts and vectors also plays a role in the establishment of an emerging pathogen and its subsequent prevalence and distribution. For example, WNV transmission in North America is greater in agricultural and urban areas, primarily because of the increased abundance of suitable hosts and vectors in these human-modified habitats [42,120]. Given the important role of mosquitoes in maintaining an active viral reservoir within an ecosystem, surveillance of mosquitoes for viral presence is paramount for understanding the local epidemiology of specific viruses. This understanding not only sheds light on pathogenicity and viral evolution but also provides essential data for developing targeted intervention and control strategies. Ultimately, the examination of virus-positive mosquito samples contributes to a holistic understanding of pathogen transmission, enabling the development of effective control strategies. It also allows for focused investigations into viral diversity within a population, further informing directed control measures and the development of vaccines and therapeutics. These crucial data not only enhance our comprehension of virus dynamics but also inform public health initiatives to combat and manage mosquito-borne diseases effectively.

## 5. Current Control Strategies for *Culex* Mosquitoes

Current methods of controlling *Culex*-borne diseases depend on reducing mosquito populations by targeting the adult and larval stages of mosquitoes. Adult control commonly utilises chemical insecticides like pyrethrin [121,122] and pyrethroids [123,124], which are known for their safety in terms of the absence of toxicity to vertebrates but pose risks to non-target invertebrates and aquatic organisms. Other commonly used insecticides include neonicotinoids, organophosphates and carbamates which have slightly higher toxicity, affecting the central nervous system of insects. In regions with stringent regulations, such as those governed by the European Parliament resolution 2002/2277, ground-level spray is preferred over aerial dispersal of insecticides, which is strictly forbidden. However, authorities do grant emergency exceptions to conduct aerial treatments during large-scale epidemics. For example, during the WNV outbreaks, experimental aerial applications in Greece using helicopters equipped with ultra-low volume nozzles were undertaken recently to spray pyrethroids like deltamethrin and d-phenothrin [124,125]. Larval control methods are mainly based on different formulations of microbial larvicides containing *Bacillus thuringiensis israelensis* (Bti) and chemical ingredients such as s-methoprene, diflubenzuron, pyriproxyfen, triflumuron, and Spinosad [125,126,127,128,129,130]. Microbial larvicides are employed to treat breeding sites, such as stagnant water bodies and containers, in an effort to limit the emergence of adult mosquitoes. Current vector control methods have been found to be effective in lowering mosquito populations in an endemic area. However, the challenges related to developing chemical resistance in mosquitoes and environmental safety concerns require ongoing innovative research to improve the effectiveness and sustainability of these interventions [131,132,133,134,135,136].

## 6. *Wolbachia*-Based Innovative Approach for *Culex* Mosquito Control

Novel *Wolbachia*-based control holds great potential as a long-term, sustainable, and environmentally friendly method for reducing vector-borne diseases. It has been effectively utilised in the field for a decade to prevent the spread of dengue in the communities by releasing *Wolbachia*-infected *Aedes* mosquitoes. A similar approach could be developed to tackle *Culex*-borne diseases. *Wolbachia* was initially identified within the gonads of *Culex pipiens* mosquitoes and belongs to the order *Rickettsiales* [137]. *Wolbachia* lineages are taxonomically organised into genetically distinct monophyletic clades termed “supergroups”, A-S [138,139].

*Culex pipiens* mosquito complexes naturally harbour a number of *Wolbachia* (*w*Pip) strains (see Table 2). Within the *Wolbachia* B supergroup, *w*Pip strains are categorised into five genetically distinct groups, designated as *w*Pip-I to V [138,140,141,142]. *w*Pip-I *Wolbachia* strains are distributed across sub-Saharan Africa, South America, Turkey, and Southeast Asia. On the other hand, *w*Pip-II groups are primarily found in Western Europe and Turkey, and *w*Pip-III strains are mostly located in North America. The occurrence of *w*Pip-IV group strains is sporadic, encompassing territories in Turkey, Europe, North Africa, and Asia, whereas *w*Pip-V strains are prevalent in Asia [142,143,144].

More than 40% of terrestrial arthropods are naturally infected with the *Wolbachia* [145]. The relationship between *Wolbachia* and its arthropod host ranges from mutualistic to parasitic depending on the host and *Wolbachia* strain combination (see [4]). In a parasitic association with the host, *Wolbachia* alters host reproductive processes to promote its own vertical transmission within host populations. One notable mechanism through which reproductive alteration is exhibited is the CI phenotype. CI phenotype can be classified into unidirectional CI and bidirectional CI. Unidirectional CI occurs when *Wolbachia*-infected males mate with uninfected females, resulting in reduced embryonic viability due to incompatibility between the modified sperm and the uninfected egg. However, viable embryos are produced if infected females mate with uninfected males. In Bidirectional CI, mating between individuals infected with different incompatible *Wolbachia* strains leads to failed embryonic development [146]. The level of CI can vary from weak to strong depending on the combination of the host and *Wolbachia* strains. For example, the *w*Mel *Wolbachia* strain causes weak CI in its native host, *Drosophila melanogaster* [147], but it leads to strong CI when introduced into a new host such as *Drosophila simulans* or *Ae. aegypti* via embryonic microinjection [148]. *w*Ri *Wolbachia* strain induces strong CI in the host *D. simulans* compared to the *w*No and *w*Ha *Wolbachia* strains, demonstrating the expression of variable CI levels in the same host due to different *Wolbachia* strains [149]. Other factors contributing to CI strength variability include *Wolbachia* density in male reproductive tissues [150], male age [151,152], and temperature [153,154]. In addition to the CI phenotype, *Wolbachia* has also been found to prevent its host from acquiring human pathogenic viral infections transmitted by arthropods (see [155]). *Wolbachia*-induced CI and virus-blocking phenotypes have formed the basis for current population suppression and replacement frameworks targeting *Aedes*-borne diseases [5,6,7,8,10,11,13,14,156,157,158]. There has been a higher degree of public acceptance toward the implementation of *Wolbachia* in field applications compared to alternative mosquito release methods, such as genetic modification or sterile insect techniques (SIT) employing mosquito irradiation. This heightened receptivity could be due to the natural occurrence of *Wolbachia* in insects [159].

**Table 2:** Natural *Wolbachia* infection in *Culex* species.

	Species	Geographical Location	*Wolbachia* Status	Reference
1.	*Cx. quinquefasciatus*	USA, Cambodia, France, Thailand, Mexico, Republic of Cape Verde, Brazil, Italy, Cuba, Malaysia, Indonesia, Singapore, Argentina, West Indies, French West Indies, Philippines, Turkey, Pakistan, Sri Lanka, India, French Polynesia, Martinique, Taiwan, Russia, Colombia, Iran, South Africa, Benin, Australia, Africa, Madagascar, Mauritius, Comoros, China, Guyana, Venezuela, Costa Rica, Puerto Rico, Haiti	+	[140,141,142,144,160,161,162,163,164,165,166,167,168,169,170,171,172,173,174,175,176,177,178,179,180,181,182,183,184,185,186,187,188,189,190,191,192,193,194,195,196,197,198,199]
2.	*Cx. molestus*	France, Sweden, UK, Australia, Tunisia, Taiwan, Russia, China, Germany, Lebanon, Belgium, Netherlands, Spain	+	[142,144,166,173,183,187,188,190,191,200,201,202]
3.	*Cx. pipiens*	Cape Verde, Leuven, Sweden, UK, Tunisia, Iran, UK, China, USA, Turkey, Algeria, Brazil, Morocco, Russia, La Reunion Island, Cyprus, Germany, Italy, Portugal, Canada, Algeria, France, South Africa, Israel, Spain, Greece, Netherlands	+	[88,141,142,143,160,166,177,178,183,187,190,197,200,203,204,205,206,207,208,209,210,211,212,213,214,215,216,217,218,219,220,221,222,223,224,225]
4.	*Cx. tigripes*	Cape Verde	+	[160]
5.	*Cx. thalassius*	Cape Verde	−	[160]
6.	*Cx. gelidus*	China, Thailand, India	+	[161,184,185,198,199]
7	*Cx. gelidus*	Sri Lanka	−	[181]
8	*Cx. tritaeniorhynchus*	China, Sri Lanka, Thailand	−	[161,181,198,199]
9	*Cx. modestus*	Belgium	−	[200]
10	*Cx. torrentium*	Belgium, Russia	−	[190,200]
11	*Cx. conservator*	Brazil	−	[164]
12	*Cx. spp*	Thailand, Egypt	+	[226,227]
13	*Cx. theileri*	Iran, Portugal	+	[206,219]
14	*Cx. theileri*	Iran	−	[192]
15	*Cx. restuans*	USA	+	[218]
16	*Cx. vishnui*	Malaysia, Singapore, India, Thailand, China	+	[167,169,184,185,198,228]
17	*Cx. pseudovishnui*	Malaysia, Singapore	+	[167,169]
18	*Cx. pseudovishnui*	India, Thailand	−	[184,185]
19	*Cx. sinensis*	Malaysia	+	[167]
20	*Cx. sinensis*	Thailand	−	[199]
21	*Cx. triataeniorhynchus*	China, Singapore, Thailand	+	[169,198,209]
22	*Cx. triataeniorhynchus*	Madagascar, Taiwan, Thailand, India	−	[184,185,188,229]
23	*Cx. sitiens*	Singapore, Thailand, India	+	[169,184,185,199]
24	*Cx. stigmatosoma*	USA	+	[210]
25	*Cx. stigmatosoma*	USA	−	[203]
26	*Cx. torrentium*	Germany	−	[211]
27	*Cx. perexigus*	Turkey	−	[178]
28	*Cx. tarsalis*	USA, Canada	−	[203,221,230,231]
29	*Cx. bitaeniorhynchus*	Singapore, Madagascar, Thailand, India	−	[169,184,185,199,229]
30	*Cx. bitaeniorhynchus*	Thailand	−	[198]
31	*Cx. brevipalpis*	Singapore	−	[169]
32	*Cx. brevipalpis*	Thailand	+	[185,199]
33	*Cx. nigropunctatus*	Singapore, Thailand	−	[169,185,199]
34	*Cx. antennatus*	Madagascar	+	[229]
35	*Cx. decens*	Madagascar	+	[229]
36	*Cx. duttoni*	Madagascar	+	[229]
37	*Cx. giganteus*	Madagascar	−	[229]
38	*Cx. poicilipes*	Madagascar	−	[229]
39	*Cx. annulirostris*	Sri Lanka	−	[181]
40	*Cx. mimulus*	Sri Lanka, Thailand	−	[181,185,199]
41	*Cx. murrelli*	Taiwan	+	[188]
42	*Cx. mimeticus*	Thailand	−	[185,199]
43	*Cx. mimeticus*	Singapore	+	[188]
44	*Cx. whitmorei*	Thailand	−	[185]
45	*Cx. whitmorei*	Thailand	+	[199]
46	*Cx. fuscancs*	Thailand, Taiwan	−	[185,188,199]
47	*Cx. pallidothorax*	Thailand	−	[185]
48	*Cx. pallidothorax*	Taiwan, Thailand	+	[185,199]
49	*Cx. fuscocephole*	Thailand, China	+	[185,198,199]
50	*Cx. eomimulus*	Taiwan	+	[188]
51	*Cx biocortus*	Taiwan	+	[188]
52	*Cx. halifaxia*	Taiwan	−	[188]
53	*Cx. okinawae*	Taiwan	−	[188]
54	*Cx. foliatus*	Taiwan	−	[188]
55	*Cx. erythrothorax*	USA	−	[214]
56	*Cx. pallens*	Japan, China	+	[190,232,233]
57	*Cx. nigripalpus*	USA	+	[233]
58	*Cx. salinarius*	Canada	+	[221]

## 7. Opportunities and Strategies for *Culex* Population Suppression Using *Wolbachia*

Population suppression framework utilising *Wolbachia* is also commonly known as the incompatible insect technique (IIT). IIT aims to provide a species-specific sustainable solution for population suppression by mass release of *Wolbachia*-infected male mosquitoes. This strategy is based on disrupting the chances of successful mating, leading to non-viable embryo production. In IIT field trials, both the unidirectional and bidirectional CI modes are utilised depending on the presence or absence of *Wolbachia* infection in the targeted population. In the case of *Ae. aegypti*, wild populations are *Wolbachia*-free, and population suppression is achieved typically through the release of males transinfected with single *Wolbachia* strains (*w*AlbB) from the laboratory, which shows a unidirectional CI pattern [6,157]. However, in the case of *Ae. albopictus* mosquitoes, both single (*w*Pip) [5,8] and triple (*w*Pip*w*AlbA*w*AlbB) infections [13] of *Wolbachia* have been trialled in the field as mosquitoes are naturally infected with two strains of *Wolbachia*: *w*AlbA and *w*AlbB. Single-strain infected *Ae. albopictus* males were created by removing the native infection of *Wolbachia* using an antibiotic and introducing the new incompatible strain *w*Pip obtained from *Cx. pipiens* [234]. A bidirectional crossing pattern is observed when these mosquitoes are mated with the wild population harbouring natural infection. In the case of triple-*Wolbachia* infected *Ae. albopictus* mosquitoes, a new *w*Pip *Wolbachia* strain, were directly added to naturally infected mosquitoes via embryonic microinjection [13]. The crossing pattern in this triple-*Wolbachia*-infected mosquitoes with wild mosquitoes is similar to that seen with unidirectional CI.

Like *Ae. albopictus* mosquitoes, *Culex* mosquitoes are naturally infected with *w*Pip *Wolbachia* strains. Historically, the diversity of *w*Pip within naturally infected *Culex* mosquito populations has been effectively utilised for IIT field trials. The first IIT trial was undertaken in 1966, using naturally *w*Pip-infected *Cx. p. fatigans* males to successfully control filariasis in Myanmar [235]. Naturally *w*Pip-infected *Cx. quinquefasciatus* males were similarly utilised in Delhi, India, in 1973 for mosquito population control [236]. Other instances of tested incompatibilities in semi-field conditions include the use of naturally *Wolbachia*-infected males of *Cx. pallens* species in China [232], *Cx. quinquefasciatus* in La Réunion Island [196,237], and a *w*Pip-IV strain from Istanbul against *w*Pip-I-infected female *Cx. quinquefasciatus* mosquitoes [196]. Apart from utilising natural *w*Pip infections, the use of embryonic microinjection has led to the successful introduction of several novel, artificial *Wolbachia* infections. An artificial infection, originally sourced from the *Aedes albopictus* mosquito into *Cx. quinquefasciatus* established a *w*AlbB single infection [238] and a *w*Pip*w*AlbA superinfection [238]. Both *w*AlbB and *w*Pip*w*AlbA superinfections induced CI, thus highlighting their potential for use in population suppression and expanding on the existing arsenal of *Wolbachia* strains that could be utilised in the field [238,239].

The genotypic diversity of *Wolbachia* strains (see Section 6) induces complex CI phenotype on the host *Cx. pipiens* complex. For example, intra-group interactions typically result in host compatibility, with occasional exceptions, while inter-group interactions often lead to embryonic mortality [143,186,191,216,217,223]. Systemic screening of *Culex* species for the presence of *Wolbachia* and characterisation of the CI phenotype effect on the host would help identify novel *Wolbachia* strains. Variable levels of CI phenotype from weak to strong have been observed depending on the *Wolbachia* strain and host combination. For instance, the *w*Au *Wolbachia* strain induces strong pathogen-blocking in *Aedes* mosquitoes but has an absence/non-detectable level of CI in both host *Ae. aegypti* and *D. simulans* [240]. Understanding the CI phenotype exhibited by these newly identified *Wolbachia* strains in their native and new *Culex* hosts (infection achieved through transinfection) would be crucial for designing IIT or population replacement trials based on these *Wolbachia* strains similar to *Ae. aegypti* and *Ae. albopictus*. Furthermore, investigation of CI effects of extensively characterised *Wolbachia* strains like *w*Ri and *w*Mel through transinfection of *Cx. quinquefasciatus* and other *Culex* vectors (see Table 1) would help elucidate the broader applicability of these strains in the IIT trial of *Culex* mosquitoes. During the IIT approach, releasing *Wolbachia*-infected *Culex* males would present several advantages compared to other existing methods. Since males do not engage in biting behaviour, any intervention is unlikely to cause an increase in biting rates post-release, thus enhancing its acceptability within communities. Further, unlike SIT methods, this approach does not introduce fitness costs that can reduce male mating competitiveness, potentially improving the efficacy of population suppression efforts [159].

Similar to the SIT approach, the IIT trial of *Culex* mosquitoes would require factory-scale production of mosquitoes and may face a few challenges that can slow down progress. A robust protocol for laboratory colonisation, mass-rearing, and continuous egg production of a targeted *Culex* species would be required. Further, a reliable sex-sorting system would be crucial as the inadvertent release of females could result in the propagation of *Wolbachia* within the population, resembling a replacement strategy rather than achieving population suppression. A hybrid approach combining IIT and SIT has been used previously to address the challenges associated with female escapees during sex sorting to avoid the accidental release of *Wolbachia*-infected females in field trials [13]. Exposure of *Wolbachia*-infected mosquitoes with low-level irradiation sterilises females while leaving males mostly unaffected. In some of the recent IIT trials, automatic machine learning-based sex sorters have been used to separate males efficiently [157,158,241]. The development of a similar sex-sorting approach for *Culex* mosquitoes would be beneficial.

## 8. *Wolbachia*-Derived CI Gene Editing for Population Control of *Culex* Mosquitoes

The genetic mechanism underlying *Wolbachia*-induced cytoplasmic incompatibility (CI) stems from a pair of closely linked and co-evolving genes, commonly referred to as *cifA* and *cifB* [242,243,244,245]. Within *w*Pip *Wolbachia* strains, multiple pairs of *cif* gene variants coexist, resulting in complex patterns of mating incompatibility between strains [212,246,247,248]. These *cif* genes exhibit significant sequence divergence, with homologs classified into five phylogenetic clusters (Types I–V) [246]. Experimental recapitulations of CI have been accomplished through transgenic expression of these *cif* genes in flies [13,236,237,241,249]. *cifA* expression in the ovaries of flies has been shown to rescue CI. Conversely, simultaneous expression of both *cifA* and *cifB* in the male testes appears to be necessary for inducing this phenotype. Recently, successful simulation of CI has been achieved by expressing *cif* genes from the *w*Pip *Wolbachia* strain in *Anopheles gambiae* mosquitoes [250] and the *w*AlbB *Wolbachia* strain in *Ae. aegypti* [251]. In both mosquito species, co-expression of *cifA* and *cifB* in testes triggered CI-like sterility, which was countered by maternal *cifA* expression, thus reproducing the pattern of *Wolbachia*-induced CI [157,158]. This development suggests the applicability of such CI gene-based methods for suppressing the population of *Culex* mosquitoes in the field.

## 9. Replacement of Wild *Culex* Population with *Wolbachia*-Infected Mosquitoes to Block Pathogen Transmission

*Wolbachia* infection has previously been shown to confer protection against pathogenic viruses [155,252], malarial parasites [252,253] and filarial nematodes [254]. CI characteristics, together with virus protection, have formed the basis for a promising *Wolbachia*-based population replacement framework targeting *Aedes*-borne diseases. In this framework, the strategy involves releasing *Wolbachia*-infected males and females into the intended wild population. Due to unidirectional CI, female mosquitoes infected with *Wolbachia* have a reproductive advantage over their wild-type counterparts, thus allowing for the natural spread of the *Wolbachia* throughout the target population, resulting in high infection frequency [255,256]. Furthermore, female mosquitoes infected with *Wolbachia* have a significantly lower capacity for transmitting viruses to humans, which subsequently leads to the reduction or possibly even elimination of diseases from endemic regions [256].

Most of the studies investigating *Wolbachia*’s ability to inhibit pathogens show a reduction in pathogen infection or transmission facilitated by *Wolbachia*, such as with WNV in *Cx. quinquefasciatus* [171,257]; however, there are exceptions. Seven days after infection, *Cx. tarsalis* infected with the transient *w*AlbB *Wolbachia* strain had a notably higher WNV infection rate compared to controls without *Wolbachia*. However, no significant differences were observed in infection, dissemination or transmission rates between the *Wolbachia*-positive and negative control at 14 days post-infection [231]. Another study in the *Cx. tarsalis* by the same laboratory found that RVFV titres had a weak negative correlation with *w*AlbB *Wolbachia* density, implying a slight suppression of RVFV replication [230]. A more recent study assessing the effect of natural and stable transfection of *w*Pip and *w*AlbB *Wolbachia* strains into *Cx. quinquefasciatus* on the transmission of avian malaria found no obvious impact on *Wolbachia* infection [239].

Multiple parameters, including virus serotype, virus titer, *Wolbachia* strain and mosquito genetic background, influence *Wolbachia’*s blocking of viruses in mosquitoes [258]. Higher *Wolbachia* density and the infection of mosquitoes’ somatic tissues have also been associated with virus-blocking [259]. Further, the novelty of the *Wolbachia*-host association also plays a role, with differential blocking levels observed between newly transferred and native *Wolbachia* variants in various mosquito species [260,261]. Future studies are warranted to investigate the potential of *Wolbachia* in blocking *Culex*-transmitted viruses such as JEV, WNV, Ross River Virus and MVEV in newly transinfected and naturally *Wolbachia*-infected *Culex* mosquitoes. This research would help design a *Wolbachia*-based population replacement approach, similar to the one employed for *Aedes* mosquitoes, aimed at the effective control of these diseases.

To effectively implement a replacement trial for *Culex* mosquitoes, it would be essential to first assess the prevalence of *Wolbachia* infection in the targeted population. This information will guide the selection of appropriate *Wolbachia* strains and the strategy for using mosquitoes infected with single or multiple strains of *Wolbachia*. The direction and strength of incompatibility influence the success of population replacement [262,263]. Unidirectional CI mode could be more effective in replacing the population than bidirectional CI mode as *Wolbachia* does not have to compete with other strains to spread into the population. Ideally, the selected *Wolbachia* strain for replacement trial should induce strong CI and block viruses, as not all strains can achieve both effects. In scenarios where *Culex* mosquitoes, such as *Cx. annulirostris* or *Cx. tarsalis* are not naturally infected with *Wolbachia*; introducing a novel strain is relatively straightforward, similar to strategies used for *Ae. aegypti*. However, most *Culex* mosquitoes are naturally infected with *Wolbachia*, complicating the replacement strategy. In such cases, a new *Wolbachia* strain needs to be added to create a superinfection, as done in *Ae. albopictus* or *Cx. Quinquefasciatus*, may be necessary. The main challenge of creating superinfection is that the existing native strain may outcompete the introduced strain, or the introduced strain may not establish itself effectively. Other factors need to be considered when carrying out replacement trials. The impact of mosquito release on the transmission of pathogens, particularly those for which the pathogen is blocked, is not tested before the release of *Culex* mosquitoes. Additionally, the stability of the *Wolbachia* strain under varying environmental conditions, such as temperature fluctuations, is important. For example, while infections with *w*Mel and *w*MelPop-CLA in *Ae. aegypti* are temperature-sensitive, *w*AlbB, which naturally colonises *Ae. albopictus* is more stable [264].

## 10. Modification of *Wolbachia* to Drive Desirable Novel Traits for Control of *Culex*-Borne Diseases

Field studies have revealed that after release, *Wolbachia* infection can easily become fixed within a population [156,265]. These results emphasize the possibility of using *Wolbachia* as a vector to introduce favourable phenotypes into mosquito populations for the control of diseases. However, difficulties associated with the inability to cultivate *Wolbachia* outside host cells have hindered genetic modification efforts. Recently, a 9228 bp extrachromosomal circular element, known as the pWCP plasmid, was identified in the *w*Pip *Wolbachia* strain from *Cx. pipiens* mosquitoes [173]. Computational analysis of pWCP plasmids in *w*Pip *Wolbachia* strains obtained from *Cx. quenquifasciatus*, *Cx. pipiens* and *Cx. molestus* mosquitoes inhabiting diverse geographical locations found that these plasmids are highly conserved [173]. Additionally, two other plasmids (pWALBA1 and pWALBA2) have been identified in the *w*AlbA *Wolbachia* strain obtained from *Ae. albopictus* mosquitoes, suggesting that plasmids may be more common in *Wolbachia* than previously thought [266]. Further, these plasmids might have a contribution towards *Wolbachia*’s ecology and evolution, which needs investigation in future. Plasmids found inside *Wolbachia* can serve as useful tools for reverse genetics to study *Wolbachia*’s gene function and manipulate *Wolbachia*, akin to approaches employed in the closely related *Rickettsia* genus [267]. Modified *Wolbachia* strains can subsequently be introduced into *Culex* mosquitoes via embryonic microinjection to drive desired traits aiming at controlling *Culex*-borne diseases.

## 11. Conclusions and Future Directions

*Wolbachia* is revolutionising the management and prevention of vector-borne disease. As part of an integrated approach, *Wolbachia* holds great promise and the potential to save millions of lives, particularly at a time when traditional tools are failing. Since the first field trials over 13 years ago, *Wolbachia* has demonstrated high phenotypic stability, achieved effective population suppression and maintained effectiveness in blocking transmission or in large *Aedes* populations. As such, *Wolbachia* has proven to be a safe, effective biotechnology that remains one of the lowest-risk biological control agents, with high public trust maintained in over 13 countries. There remains considerable upside to investing in and adapting the technology to prevent the spread of human pathogens in *Culex* populations. However, there are several challenges as *Culex* vector and virus ecological interactions are complex, requiring a multidisciplinary and integrated pest management approach for effective, long-term and sustainable control. Although early *Culex* studies provide examples of where *Wolbachia* can modify phenotypes in unpredictable ways, they also demonstrate the rigorous science required to adopt these technologies for safe deployment and realise the public health benefits. Opportunities exist for exploring the great potential of modifying large populations of *Culex* vectors to prevent the spread of pathogens such as JEV or WNV in human populations or with avian malaria for conservation purposes. A greater understanding of the underlying genetic mechanisms of *Wolbachia* CI and virus-blocking phenotypes holds the potential for more targeted genetic approaches that confer the benefits of *Wolbachia* without the difficulty associated with establishing *Wolbachia* infections. In a world where vector-borne risk is increasing rapidly, the adoption of *Wolbachia* as an effective tool in the public health toolbox will be essential.
